# Diagnostic and therapeutic yields of balloon-assisted enteroscopy on different subtypes of patients with suspected small bowel bleeding

**DOI:** 10.1093/jcag/gwaf037

**Published:** 2025-12-22

**Authors:** Brendan Halloran, Jimmy Yimeng Guo, Getanshu Malik, Shawn Wasilenko, Aldo J Montano-Loza, Sergio Zepeda-Gomez

**Affiliations:** Division of Gastroenterology, University of Alberta, Edmonton, AB, Canada; Division of Gastroenterology, University of Alberta, Edmonton, AB, Canada; Division of Gastroenterology, University of Alberta, Edmonton, AB, Canada; Division of Gastroenterology, University of Alberta, Edmonton, AB, Canada; Division of Gastroenterology, University of Alberta, Edmonton, AB, Canada; Division of Gastroenterology, University of Alberta, Edmonton, AB, Canada

**Keywords:** balloon-assisted enteroscopy, obscure gastrointestinal bleeding, small bowel bleeding

## Abstract

**Background:**

Small bowel bleeding accounts for about 5%-8% of all cases of gastrointestinal bleeding. Suspected small bowel bleeding (SSBB) can be classified into occult, inactive overt, and overt. Most patients with SSBB will undergo balloon-assisted enteroscopy (BAE) for diagnosis and treatment. There are currently no recommendations from practice guidelines on what is the best approach and limited information about diagnostic and therapeutic yields for each subtype of SSBB.

**Aims and Methods:**

We aimed to investigate the diagnostic and therapeutic yields of BAE in the 3 subtypes of patients with SSBB by performing a retrospective analysis of all patients that underwent BAE for this diagnosis at the University of Alberta Hospital in a 5-year period. We also aimed to identify other factors that could influence diagnostic and therapeutic yields.

**Results:**

The overall diagnostic and therapeutic yields of BAE for SSBB were 66% and 51%, respectively. When stratified by subtypes of SSBB, the diagnostic yield for occult, inactive overt, and active overt SSBB were reported to be 61%, 67%, and 95% (*P* < .05), respectively. BAE performed within 72 hours of presentation and patients requiring transfusion within the past 12 months had a significantly higher diagnostic yield.

**Conclusions:**

Our data showed the clinical differences between the 3 subtypes of patients with SSBB and the usefulness of an appropriate and timely approach to maximize the diagnostic and therapeutic yields.

## Introduction

Gastrointestinal (GI) bleeding is a life-threatening medical emergency with an annual incidence rate for upper and lower GI bleeding of 90 per 100 000 and 33 per 100 000, respectively.^1^^,^^2^ Small bowel bleeding accounts for about 5%-8% of all cases of GI bleeding. Suspected small bowel bleeding (SSBB) is defined as bleeding from an unknown source despite initial investigations with gastroscopy and colonoscopy, of which up to 75% can be attributed to a small bowel source.^3–7^ This was previously known as Obscure GI bleeding (OGIB), which now should be reserved for patients with also negative small bowel investigations.^3^ The clinical presentation of patients with SSBB may be subdivided into 3 subtypes: active overt bleeding (patient with ongoing active bleeding symptoms), inactive overt bleeding (patient with recent signs of overt bleeding), and occult bleeding (absence of overt bleeding).

With the advent of video capsule endoscopy (VCE) and balloon-assisted enteroscopy (BAE), the diagnostic and therapeutic yields for investigation and treatment of patients with SSBB have significantly improved.^8–11^ Diagnostic yields range from 45% to 87%, while therapeutic yields range from 21% to 84%.^5^^,^^12–21^ Some patient and comorbidities factors that are associated with increased diagnostic and therapeutic yields include increased age, male sex, ongoing bleeding requiring transfusion, suspected small bowel neoplasia, and cirrhosis.^14^^,^^22^ Optimized timing between the most recent episode of overt GI bleed and small bowel investigations has been proposed to maximize diagnostic and therapeutic yields.^5^^,^^17^^,^^23^ There are currently no recommendations from existing practice guidelines on finding the most effective timing for endoscopic interventions.^3^ As such, determining and finding consensus for optimal timing of BAE in SSBB remains an ongoing question.

In this study, our primary objectives were to calculate diagnostic yield, therapeutic yield, and rebleeding rate for all clinical subtypes of SSBB based on a large single-centre cohort. Secondary outcomes were to identify optimal timing of BAE in relation to the presentation with SSBB and/or a positive finding at VCE, in relation to the primary outcomes.

## Methods

### Study population

**Figure 1:**
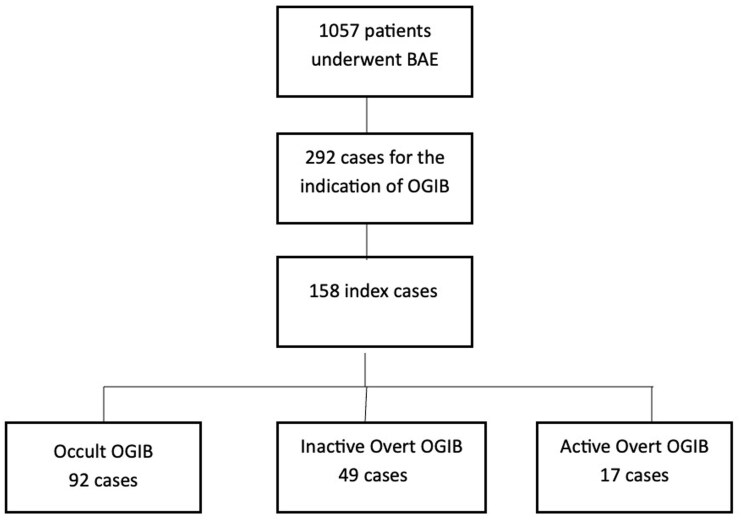
A total of 1057 BAE cases were completed, of which 292 cases were completed for the indication of obscure GI bleed. Index cases of 158 patients were included in our final analysis, which were divided into 3 subcategories of overt GI bleed.

We performed a retrospective review from a prospectively followed patient cohort at a high-volume, quaternary centre (University of Alberta Hospital, Edmonton, Canada) which included all adult patients who underwent BAE between January 2016 and April 2021. During the study period, a total of 1057 BAE cases were performed, of which 292 cases were completed for the indication of SSBB. To prevent against falsely elevated diagnostic and therapeutic yields in subsequent BAE, only index cases of 158 patients were included in our final analysis (Figure 1). Patients were identified from a prospectively maintained database of balloon-assisted enteroscopies performed at our Institution. Mislabelled procedures and patients with unavailable endoscopic reports were excluded. Patients with established Crohn’s disease and a history of small bowel bleeding were also excluded from the study.

### Objectives

The primary objectives were to calculate the diagnostic and therapeutic yields of patients who underwent BAE for evaluation of SSBB. Secondary objectives were to assess for association between timing of BAE from an episode of overt GI bleeding and small bowel imaging with clinical outcomes including diagnostic yield, therapeutic yield, and rebleeding rate. In addition, we aimed to identify patient factors that could be associated with the above-mentioned clinical outcomes.

### Terminology

SSBB is classified into occult, inactive overt, and active overt bleeding. Occult GI bleeding refers to cases of iron deficiency anaemia without overt GI bleeding. In overt GI bleeding, patients have melena or haematochezia with suspicion to have a small bowel source of blood loss. Inactive overt bleeding refers to cases of patients with history of overt GI bleeding that has clinically subsided for more than 24 hours at the time of evaluation. Lastly, active overt GI bleeding refers to cases with signs of ongoing active bleeding, at the time of or within 24 hours of evaluation. Diagnostic yield was defined as the proportion of procedures with a positive endoscopic finding, whereas therapeutic yield was defined as the proportion of procedures during which a therapeutic intervention was performed.^24^^,^^25^ A therapeutic intervention was defined as haemostatic injection, argon plasma coagulation, placement of haemoclips, or a combination of these modalities or another endoscopic intervention aimed to perform haemostasis.

### BAE procedure

All BAE procedures were performed using either the single-balloon (SBE) or double-balloon (DBE) systems (SIF-Q180, Olympus, EN-450T, FUJIFILM, Japan), by 3 high-volume endoscopists with specialized training in BAE (S.W., B.H., S.Z.-G.). Patients referred for BAE would be required to have recent gastroscopy and a complete colonoscopy with satisfactory preparation and endoscopic views. Over 90% of cases underwent small bowel imaging including VCE, CT enterography (CTE), or MR enterography (MRE) prior to BAE. For oral BAE, the patient was required to have at least 8 hours of fasting prior to the procedure and consume 2 L of polyethylene glycol-based solution. For rectal BAE, the patients were prepared with 6 L of polyethylene glycol-based solution using split dosing method, similar to the preparation of a colonoscopy. All cases were performed with anaesthesia support, and orotracheal intubation was performed on patients who underwent oral route BAE. The route of BAE was decided based on preceding VCE and/or CTE/MRE. If the location of suspected culprit lesion was less than 70% of the total small bowel transit time (SBTT), which is the time the VCE spending in the small bowel between the stomach and cecum, an oral BAE was performed.^26^ In cases where prior imaging was unavailable, the BAE route was selected based on patient history and physical examination; melena stools would usually prompt initial investigation with oral BAE, whereas maroon stools or haematochezia would prompt a rectal BAE.

### Data collection

Patient characteristics, including age, sex, comorbidities (coronary artery disease, chronic renal disease, ongoing haemodialysis, aortic stenosis, history of cardiac valve replacement or left ventricular assist device placement, atrial fibrillation, DVT, pulmonary thromboembolism, liver cirrhosis, hereditary haemorrhagic telangiectasia, and diabetes), medication use (warfarin, direct oral anticoagulants, anti-platelet agents, and iron supplement), and rebleeding rate were documented. Periprocedural variables including indication, type of SSBB (occult, inactive overt, active overt), prior endoscopic and imaging results, endoscopic findings, therapies applied, and complications were recorded via the endoscopic report.

### Statistical analysis

Continuous variables were reported as mean with standard deviation and the categorical values were presented as frequency and percentages. The differences in the mean of the baseline characteristics of the type of GI bleeding, occult, inactive overt, and active overt were compared using the unpaired *t*-test, and the relations between categorical variables were confirmed using Fisher’s exact test. Features associated with diagnostic yield was investigated using univariate and multivariate logistic regression. All tests were 2-sided, and a *P*-value < .05 was considered statistically significant. Factors with a *P*-value < .10 in the univariate analysis were incorporated into the final multivariate model.

Overall rebleeding-free survival estimates and survival probabilities were obtained by applying the Kaplan-Meier curves and the comparison between survival curves was conducted by means of the Log Rank (Mantel-Cox) test. Patients who were missed throughout follow-up and patients who were constantly monitored in the clinic were censored at the time of study termination. Statistical analyses were conducted using SPSS (SPSS for Windows, version 28.0, SPSS, Chicago, IL) and a *P* alue less than .05 was a statistically significant difference.

### Ethical considerations

This study was approved by the Alberta Research Information Services Health Research Ethics Board—Health Panel (Pro00111003).

## Results

### Study population

In total, 1057 cases of BAE were identified within the study period with 292 cases performed for OGIB. Analyses were completed for 158 index cases: 92 occult SSBB cases (58%), 49 inactive overt cases (31%), and 17 active overt cases (11%). The mean age was 65 ± 14.4 years, 64 patients were female (41%), and 115 antegrade BAE (73%) were completed. The DBE system was used for 116 cases (73.4%). Complete baseline characteristics of this study cohort can be found in Table 1. Of the total procedures, 144 cases were preceded by small bowel imaging (91%): 117 were preceded by VCE (74%), 65 cases underwent prior CTE (41%), and 36 cases underwent both (22%). All patients had negative findings on esophago-gastro-duodenoscopy (EGD) and colonoscopy prior to BAE.

**Table 1:** Demographics and different variables among the 3 subtypes of patients with SSBB.

Variables	Occult (*n* = 92)	Inactive overt (*n* = 49)	Active overt (*n* = 17)	Total (*n* = 158)
**Age, years (SD)**	65 ± 13.5	63 ± 15.5	68 ± 16.14	65 ± 14.4
**Female (sex)**	44 (48)	16 (33)	4 (24)	64 (41)
**Comorbidities**				
**Coronary artery disease**	21 (23)	12 (25)	8 (47)	41 (26)
**Chronic kidney disease**	6 (7)^a^	3 (6)^b^	6 (35)^a,b^	15 (10)
**Haemodialysis**	1 (1)	1 (2)	0	2 (1)
**Aortic stenosis**	5 (5)	4 (8)	0	9 (6)
**Cardiac valve replacement**	0^c^	0^d^	3 (18)^c,d^	3 (2)
**Left ventricular assist device**	1 (1)	0	0	1 (1)
**Atrial fibrillation**	5 (5)^e^	8 (16)^f^	11 (65)^e,f^	24 (15)
**Deep vein thrombosis**	1 (1)	0	0	1 (1)
**Pulmonary embolism**	2 (2)	1 (2)	0	3 (2)
**Cirrhosis**	6 (7)	5 (10)	3 (18)	14 (9)
**Hereditary haemorrhagic telangiectasia**	4 (4)	2 (4)	1 (6)	7 (4)
**Diabetes**	22 (24)	14 (29)	6 (35)	42 (27)
**Medications**				
**Warfarin**	4 (4)	2 (4)	3 (18)	9 (6)
**Aspirin**	17 (19)	10 (20)	6 (35)	33 (21)
**Ticagrelor**	1 (1)	1 (2)	0	2 (1)
**Clopidogrel**	4 (4)	6 (12)	2 (12)	12 (8)
**Dual antiplatelet therapy**	3 (3)	1 (2)	1 (6)	5 (3)
**Dabigatran**	0	0	0	0
**Apixaban**	2 (2)^g^	2 (4)^h^	4 (24)^g,h^	8 (5)
**Rivaroxaban**	6 (7)^i^	3 (6)^j^	4 (24)^i,j^	13 (8)
**Direct oral anticoagulants**	8 (9)^k^	5 (10)^l^	8 (47)^k,l^	21 (13)
**Tinzaparin**	0^m^	0	1 (6)^m^	1 (2)
**Iron supplement**	52 (57)	22 (45)	5 (29)	79 (50)
**Anterograde balloon-assisted enteroscopy**	63 (69)	38 (80)	13 (77)	115 (73)
**Prior imaging**				
**Video capsule endoscopy**	69 (75)	37 (76)	11 (65)	117 (74)
**CT enterography**	41 (45)	20 (41)	4 (24)	65 (41)

Numbers in parentheses are percentages.

Significantly different from each other at the level of ^a,b,c,d,e,f^*P* < .001, ^g,h^*P *= .001, ^i,j,k,l^*P *= .05, and ^m^*P *= .02.

### Primary outcomes

The overall diagnostic yield of BAE was 66% with positive endoscopic findings reported in 105 cases. When stratified by subtypes of SSBB, the diagnostic yield for occult, inactive overt, and active overt SSBB were reported to be 61%, 67%, and 95% (*P* < .05), respectively. The findings included vascular lesions (*n* = 67, 42%), ulcerations and erosions (*n* = 17, 11%), strictures with ulceration (*n* = 13, 8%), neoplastic lesions (*n* = 8, 5%), and small bowel diverticular bleed (*n* = 6, 4%). Lesions were most commonly found in the proximal jejunum (*n* = 68, 43%), followed by distal duodenum (*n* = 22, 14%), ileum (*n* = 17, 11%), and mid-distal jejunum (*n* = 2, 8%).

The overall therapeutic yield was 51% with endoscopic interventions performed in 81 cases. Stratified by subtypes of SSBB, the therapeutic yield of occult, inactive overt, and active overt OGIB was reported to be 54%, 41%, and 65%, respectively (NS). Table 2 shows the diagnostic and therapeutic yields of the different subtypes of patients with SSBB. Interventions performed across all subtypes include argon plasma coagulation (*n* = 61, 75%), haemoclip application (*n* = 41, 51%), epinephrine injection in combination with other therapy (*n* = 6, 7%), and epinephrine injection as monotherapy (*n* = 1, 1%). Haemoclip application and epinephrine injection were employed more often in patients presenting with active overt bleeding (*P* < .001).

**Table 2:** Diagnostic and Therapeutic yields of different subtypes of patients with SSBB.

	Occult	Inactive overt	Active overt	Total
**Total**	92	49	17	158
**Positive findings** (*n*)	56	33	16	105
**Diagnostic yield (%)**	61^a^	67^b^	94^a,b^	66
	**Occult**	**Inactive overt**	**Active overt**	**Total**
**Total**	92	49	17	158
**Interventions**	50	20	11	81
**Therapeutic yield (%)**	54	41	65	51

Significantly different from each other at the level of ^a,b^*P *= .05.

The overall rebleeding rate for patients presenting with overt bleeding was 45% (*n* = 30). Twenty-eight (28) patients were sent for a VCE study post-procedure (18%), while 32 patients underwent repeat BAE (20%). Medical management was initiated for 31 patients (20%) post-BAE: octreotide (*n* = 17, 11%), bevacizumab (*n* = 2, 1.3%), thalidomide (*n* = 1, 0.6%), as well as IV iron infusions, and new biologic start for small bowel Crohn’s disease. Surgical management and/or interventional radiology (IR) therapy was undertaken in 17 cases (11%). BAE findings ultimately led to a change in clinical pathway for 33% of all cases.

### Secondary outcomes

#### Timing of endoscopy in relation to diagnostic yield, therapeutic yield, and rebleeding rate

Increased diagnostic yield was found when BAE was performed within 72 hours of an overt bleeding episode (*P* = .05) compared to more than 72 hours (Table 3). No increase in diagnostic yield among the different group subtypes was seen at 7 or 14 days after an episode of overt bleeding.

**Table 3:** Timing of endoscopy in relation to diagnostic and therapeutic yields and re-bleeding rates in patients with SSBB.

Timing of balloon-assisted enteroscopy after last episode of overt bleeding	≤ 72 hours	> 72 hours	Chi-squared
*n*	17	49	0.05
**Positive findings**	16	33	
**Diagnostic yield (%)**	94	67	
**Therapy applied**	11	20	0.1
**Therapeutic yield**	65	41	
**Rebleeding** (*n*)	7	22	1.0
**Rebleeding (%)**	41	45	

#### Factors associated with active overt GI bleed and increased diagnostic yield

Presentations of active overt bleeding were significantly associated with known chronic kidney disease (CKD) (*P* < .001), cardiac valve replacement (*P* < .001), atrial fibrillation (*P* < .001), current DOAC use (*P* = .05), and therapeutic tinzaparin treatment (*P* = .02) (Table 1).

The association between patient and endoscopic factors (basic demographics, disease factors, and timing of BAE) and diagnostic yield was assessed using univariate logistic regression analysis. We found a statistically significant association between transfusion requirement within the last 12 months and higher diagnostic yield (OR: 2.32; 95% confidence interval [CI], 1.12-4.84; *P* = .02). Additionally, a trend toward significance was observed for association between DOAC use (OR: 3.78; 95% CI, 0.84-17.00; *P* = .08) and active overt GI bleeding with a higher diagnostic yield (OR: 1.13, 95% CI, 0.42-3.06; *P* = .08) (Table 4).

**Table 4:** Association of Diagnostic Yield with different variables in patients with SSBB.

Features	Univariate		
	OR	95% CI	*P*
**Sex, male**	0.72	0.35-1.48	.4
**Age**	1.02	0.99-1.04	.2
**Coronary artery disease**	1.33	0.58-3.11	.5
**Chronic kidney disease**	2.44	0.53-11.30	.3
**Aortic stenosis**	0.69	0.16-2.87	.6
**Atrial fibrillation**	1.91	0.61-5.95	.3
**Cirrhosis**	1.31	0.35-4.97	.7
**Diabetes**	1.40	0.60-3.24	.4
**ASA**	1.12	0.46-2.73	.8
**Rivaroxaban**	2.02	0.43-9.54	.4
**DAPT**	0.22	0.04-1.37	.1
**DOAC**	**3.78**	**0.84-17.00**	**.08**
**Occult GI bleeding**	0.98	0.48-2.02	.9
**Inactive overt GI bleeding**	0.62	0.29-1.29	.2
**Active overt GI bleeding**	**6.34**	**0.81-49.38**	**.08**
**Days after last episode SSBB**	0.99	0.99-1.00	.3
**Days after video capsule endoscopy**	1.001	0.99-1.003	.5
**Positive finding video capsule endoscopy**	1.01	0.46-2.21	.9
**Positive findings CT enterography**	1.13	0.42-3.06	.8
**Active bleeding**	3.74	0.46-30.15	.2
**Transfusion within last 12 months**	**2.32**	**1.12-4.84**	**.02**

Abbreviations: ASA, acetylsalicylic acid; DAPT, Dual anti-platelet therapy; DOAC, Direct oral anticoagulant;GI, gastrointestinal; OGIB, obscure GI bleeding.

#### Survival analysis and safety

Using Kaplan-Meier survival analysis, the overall rebleeding free survival for all patients at 1 year and 5 years was calculated to be 98% and 68%, respectively. No major complications including major bleeding requiring admission, perforation, or death were identified within the study period or subsequent follow up. Two patients experienced transient increase in oxygen requirements post-procedure, which resolved spontaneously.

## Discussion

In this study, 158 patients who underwent index BAE for the investigation of SSBB, the subgroup type and the timing of endoscopic assessment were associated with a significantly higher diagnostic yield among other factors. Presentations with active overt bleeding, as well as patients requiring transfusion within the past 12 months led to a higher diagnostic yield compared to those with occult and inactive overt bleeding and those without transfusion requirements. In the active and inactive overt GI bleeding patients, BAE performed within 72 hours of presentation was found to have a higher diagnostic yield.

### Primary endpoint

We found an overall diagnostic yield of 66% in our patient cohort, which is in keeping with previously published data ranging from 47 to 87%.^5^^,^^17^^,^^19–22^^,^^25^^,^^27–29^ Our reported therapeutic yield of 51% is also congruent with current literature that ranges from 43% to 84%.^7^^,^^22^ The highest diagnostic and therapeutic yield (95% and 65%) was seen in the active overt bleeding subgroup, confirming that BAE is most effective in the identification and treatment of culprit lesions in the setting of an acute bleeding episode. It should be noted that this was on index BAE and did not capture further investigations and endoscopic procedures.

### Secondary endpoints

We found a statistically significant increase in diagnostic yield when BAE was performed within 72 hours after an overt bleeding episode. This corresponds with previous literature showing the benefit of performing urgent enteroscopy in the setting of SSBB with overt GI bleeding. A 2018 retrospective analysis of 70 BAE reported an increase in the diagnostic yield when urgent enteroscopy was performed within 72 hours of an active bleed compared to delayed intervention group (84.5 vs 50.0%, *P* < .01).^5^ In a 2020 retrospective cohort of 33 patients, authors demonstrated that urgent BAE performed within 72 hours of an overt GI bleed led to a higher diagnostic yield (88.2 vs 59.5%, *P* = .03).^39^ We did not find a statistically significant difference in diagnostic yield when the timing of intervention happened at 7-14 days from presentation. Our study suggests that BAE should optimally be performed within the first 72 hours of an episode of overt bleeding to increase the odds of diagnosis and treatment of culprit lesions.

We found several patient factors that are associated with a higher likelihood of finding a culprit lesion. These include a history of cardiac valve replacement, atrial fibrillation, CKD, and current DOAC and therapeutic tinzaparin use. Ohmiya et al.assigned a weighted value to various comorbidities to allow for prediction of small bowel vascular disease.^31^ Dialysis-dependent CKD, peripheral vascular disease, and valvular heart disease are among some of the comorbidities that are associated with a higher risk of small bowel bleeding from vascular lesions in the small bowel. Heyde’s syndrome^40^^,^^41^ and direct oral anticoagulation use are also risk factors associated with the development of small bowel vascular lesions.^42–44^ CKD is an independent risk factor for GI bleed given its associations with development of vascular lesions including angiodysplasia.^45–51^ As such, previous studies suggested that GI bleeding in patients with CKD has worse outcomes, leads to higher mortality, and generates higher healthcare costs compared to those with normal renal function.^46^^,^^52^^,^^53^

In our study, a statistically significant association between transfusion requirements within the past 12 month and increased diagnostic yield was observed which, again, may signify that the intensity of the bleed or rapidity of presentation plays a role in diagnostic yield. Slower, gradual, and intermittent small bleedings are more difficult to identify and may result from multiple lesions and ultimately will require medical therapy (iron infusions, octreotide, etc).

A trend towards statistical significance was also observed for patients presenting with active overt GI Bleeding and current use of DOAC. A number of factors associated with increased diagnostic and therapeutic yield have been identified in previous literature, including increased age, male sex, ongoing overt bleeding requiring transfusion, cardiovascular disease, suspected small bowel neoplasia, and liver cirrhosis.^20^^,^^25^^,^^32^^,^^34^ Although we did not detect the other risk factors mentioned above, the identified factors in this study are likely associated with ongoing recurrent or intermittent bleeding from small bowel pathology and led to higher likelihood of detection.

We found a rebleeding rate of 45% in patients with overt OGIB, this is similar to our current knowledge of patients with bleeding from vascular lesions, where rates of up to 40% have been reported. This rebleeding rate is likely secondary to several factors: multiple vascular lesions responsible for bleeding episodes, culprit lesion (s) not identified on initial endoscopy, development of new vascular lesions, and non-modifiable patient risk factors. Findings of vascular lesions within the proximal small bowel potentially signal additional lesions within the distal GI tract that were not detected nor could be treated to its entirety during the index case. Furthermore, small bowel angiodysplasia frequently presents as multiple lesions and can be missed on BAE or multiple high-risk lesions may exist concomitantly (ie, Heyde’s syndrome or Hereditary Haemorrhagic Telangectasia [HHT]). Targeted treatments of the presumed bleeding lesion may not be the culprit or sole bleeding source. Finally, in the presence of non-modifiable risk factors, vascular lesions are likely to reappear which leads to recurrent bleeding. We report a 61% IDA recurrence rate in patients with occult bleeding. Small vascular lesions, often seen in occult bleeding cases may bleed intermittently compared to their larger counterparts, which is why initial BAE may not adequately prevent future rebleeding events.^30^ In our study, 34% of all patients required repeat VCE and/or BAE for this reason. Sakai et al.demonstrated that, although a single session of BAE was not sufficient in prevention of rebleeding, bleeding was ultimately controlled with repeat endoscopic treatment and/or iron replacement therapy in most cases.^30^ There were 14 patients that underwent surgical treatment after BAE identification of different sources of bleeding in small bowel such as neuroendocrine tumours (*n* = 2), Meckel’s diverticula (*n* = 3), ileocolonic resection for giant ileal and colonic vascular malformation (*n* = 1), ileal gastrointestinal stromal tumour (*n* = 1), bleeding jejunal polyp (*n* = 1), Crohn’s stricture with ulceration in SB (*n* = 2), jejunal adenocarcinoma (*n* = 1), cardiac surgery for aortic stenosis and Heyde’s syndrome (*n* = 3), and 3 patients underwent embolization through interventional radiology for arterial bleeding secondary to Dieulafoy lesions in the small bowel.

### Safety

A low complication rate (1.3%) was noted in our cohort. Two patients experienced a transient increase in oxygen requirements post procedure, likely due to sedation related hypoxemia but did not require hospitalization. Previous literature showed that diagnostic BAE can result in minor complications, affecting approximately 9.1% of patients. These include self-limiting abdominal pain, sore throat, and sedation-related effects.^33^ Other complications were seen in less than 2% of patients, which include bleeding (0.8%), perforation (0.3%), and pancreatitis (0.3%).^22^^,^^25^^,^^27^^,^^29^^,^^35^^,^^36–38^^,^^54^^,^^55^ None of these were observed in our study.

### Limitations and future directions

The major limitations of our study are retrospective design and posteriori analysis generated from previous health records and endoscopic reports. As such, selection bias was unavoidable and risk factors could only be reported as associations rather than established causations. However, all factors identified were previously reported in the literature, strengthening the validity of these observations. We reported our local experience from a single quaternary care centre in an area with a province wide electronic medical record for all inpatient encounters. Thus, a large prospectively designed study with long-term follow-up would address the above limitations. Lastly, the cases were restricted to index procedures to prevent falsely inflated diagnostic rates, which limited the sample size. Our study was meant to take an initial look at outcomes; we have yet to look at the impact of presentation, diagnosis and therapeutic intervention on rebleeding rates over time. This is a very important question and is the subject of ongoing analysis with expansion of the data collection.

## Conclusions

This is the first study to assess diagnostic, therapeutic, and rebleeding rates for BAE in all SSBB subtypes. Ultimately, our study demonstrated that BAE is an effective diagnostic and therapeutic tool and its use needs to be refined and targeted to enhance diagnostic yield and improve patient outcomes. We found a significantly improved diagnostic rate when BAE is performed within 72 hours of an episode of overt bleeding episode and in patients with ongoing active bleeding. Further to this, patients who have required transfusions also had higher diagnostic yield on BAE, suggesting that the severity of presentation is associated with a higher yield in BAE. This affirms the point that timing of BAE matters in the acute phase and that accurate identification of the SSBB subgroup is crucial for the approach and management of these patients.

## Supplementary Material

gwaf037_Supplementary_Data

## Data Availability

The data that support the findings of this study are available from the corresponding author upon reasonable request.
